# Constitutive Analysis and Microstructure Characteristics of As-Homogenized 2198 Al–Li Alloy under Different Hot Compression Deformation Conditions

**DOI:** 10.3390/ma16072660

**Published:** 2023-03-27

**Authors:** Huiyu Li, Xiwu Li, Hongwei Yan, Yanan Li, Libo Geng, Chenyang Xun, Zhihui Li, Yongan Zhang, Baiqing Xiong

**Affiliations:** 1 State Key Laboratory of Nonferrous Metals and Processes, GRINM Group Co., Ltd., Beijing 100088, China; 2 GRIMAT Engineering Institute Co., Ltd., Beijing 101407, China; 3 General Research Institute for Nonferrous Metals, Beijing 100088, China; 4 Southwest Aluminium (GROUP) Co., Ltd., Chongqing 401326, China

**Keywords:** 2198 Al–Li alloy, hot compression, processing maps, microstructure evolution

## Abstract

The 2198 Al–Li alloy has unique superiority in mechanical performance and has been extensively used in the aerospace field. In this study, the hot deformation behavior of the 2198 Al–Li alloy was investigated on a Gleeble-1500 thermomechanical simulator with a strain rate of 0.01–10 s^−1^ in the temperature range of 330–510 °C. The Arrhenius constitutive equation of the alloy was established based on the true stress–strain curves to describe the rheology behaviors during the deformation of the alloy. The processing maps under the strain of 0.2–0.8 were constructed, which indicates the efficiency of power dissipation and instability of the deformed alloy. It was found that the instability domains are more likely to occur in the regions of low deformation temperature and high strain rate, corresponding to the high Zener–Hollomon (*Z*) parameter. The microstructure evolution of the studied alloy with different *Z* parameters was characterized. Then, the dynamic recrystallization (DRX) behavior was studied by electron backscatter diffraction, and the misorientation angle of deformed specimens was analyzed. The effect of different deformation temperatures and strain rates on the microstructure of the alloy and the behavior of dislocations and precipitations were investigated by transmission electron microscopy. The results demonstrate that continuous dynamic recrystallization (CDRX) and geomatic dynamic recrystallization (GDRX) mainly occur at the deformation conditions of a low *Z* value, and discontinuous dynamic recrystallization (DDRX) is likely to occur with increasing *Z* values.

## 1. Introduction

With the increasing demand for lightweight structure materials, the Al–Li aluminum alloy has been widely used in the aerospace manufacturing industry due to its excellent mechanical properties. As one of the newly developed alloys, the 2198 Al–Li alloy shows superiority for use in aerospace structural materials, such as low density, good formability, high strength, and weight reduction effect [1,2,3,4]. The hot deformation processes, such as forging, extrusion, and rolling, are the main means to manufacture structural materials in the aerospace field. The hot compression test is an ideal method to study the thermal deformation characteristics of aluminum alloys, and the constitutive equations determined from the compression test data are widely used to describe the flow stress behavior of materials [5,6]. It is necessary to conduct the hot compression tests on the 2198 Al–Li alloy in different deformation conditions because the flow behavior significantly influences the microstructure evolution of the alloy.

It has been extensively accepted that the flow behavior and microstructure are relevant to several factors, including deformation temperature, strain rate, and total strain [7,8]. Therefore, the establishment of a constitutive equation is a necessary methodology for analyzing the hot deformation behavior and predicting the flow stress of materials. The most extensively used method for analyzing the mechanical response of materials during high-temperature plastic deformation is based on the constitutive equations related to flow stress, temperature, and strain rate. Some researchers have introduced the finite volume method into hot-working simulations to estimate changes in flow stress due to strain, temperature, and strain rate [9]. In addition, the Hansel–Spittel model is also used to simulate the stress response process of high-temperature deformation of aluminum alloys, but is more commonly used to describe the plastic deformation behavior of steel and magnesium alloys at high temperatures [10]. Generally, the predictive ability of the Arrhenius-type constitutive equation of aluminum alloys has proven to be accurate [11,12,13]. Furthermore, the processing map based on the dynamic material model (DMM) is considered a practical method for the guidance of the hot working in alloys, predicting the safety domains and instability domains and determining the deformation temperature and strain rate in reference to a relatively high efficiency of power dissipation [14,15,16]. Some researchers proved that the microstructure of the alloy deformed in the instability domains of processing maps shows obvious flow localization [17,18].

In addition, extensive research has shown that dynamic recovery (DRV) and dynamic recrystallization (DRX) play a crucial role in the microstructure evolution during the hot deformation of the alloy [19,20,21,22]. Considered to be one of the most important microstructure evolutions of materials, DRX helps to refine grains, eliminate defects, and improve the material properties and is also beneficial to reduce the anisotropy of the alloy, which is of great significance for the Al–Li alloy [23,24]. Khokhlatova et al. [25] investigated the relationship between recrystallization and anisotropy of the 1424 alloy sheets and found that the fully recrystallized structure significantly reduced the tensile property anisotropy in three directions in cold-rolled sheets. DRX of the aluminum alloy is sensitive to deformation conditions, and there have been some improvements in the study of the dynamic softening behavior of the Al–Li alloy through thermal simulation compression experiments. For example, Zhang et al. [20] suggested that continuous dynamic recrystallization (CDRX) was the main softening mechanism during high-temperature deformation, which is characterized by the increase in the grain boundary misorientation angles. They also pointed out that three types of subgrain-forming mechanisms were involved in the CDRX of the 2195 aluminum alloy, and the bamboo-like recrystallized grains caused by geomatic dynamic recrystallization (GDRX) appeared near the grain boundaries when the strain increases at high temperature. Wang et al. [26] studied the effect of the strain rate on the DRX mechanism, and the result showed that dynamic recovery (DRV) occurred at a high strain rate, while CDRX and low proportion discontinuous dynamic recrystallization (DDRX) appeared at a low strain rate. However, different types of DRX of the aluminum alloy may co-occur under certain deformation conditions [27]. Currently, there are many studies on the effects of hot deformation conditions on the flow behavior and microstructure evolution of aluminum alloys, but the 2198 Al–Li alloy is rarely involved.

In the present work, the hot deformation behaviors of the as-homogenized 2198 Al–Li alloy were investigated. The constitutive equation and processing maps were established based on the stress–strain curves to describe the effect of deformation temperature and strain rate on rheology behaviors and determine the optimal hot-working process. Deformation microstructure characteristics with different *Z* parameters were observed by comparing the variation of misorientation angles and distribution of dislocations, where the dynamic softening mechanism in the compression process was analyzed. In addition, the effect of deformation temperature and strain rate was also investigated by characterizing the microstructure.

## 2. Materials and Experimental Methods

**Figure 1:**
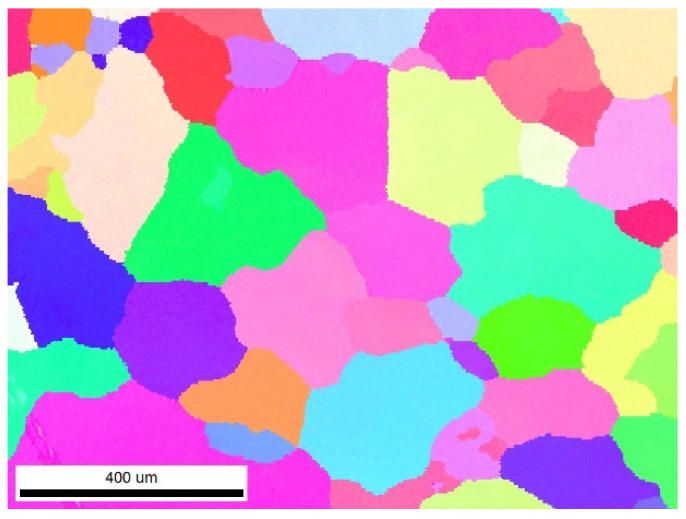
IPF map of the homogenized 2198 Al–Li alloy without compression.

The chemical composition of the studied alloy in this paper is shown in Table 1. The experimental material was manufactured by melting and casting, with a casting temperature of 710–730 °C and ingot dimensions of 400 × 1350 × 4000 mm. and then homogenized in a thermal resistance furnace at 410 °C for 16 h and 495 °C for 24 h, which was cooled in the air to room temperature. Figure 1 shows the IPF map of the undeformed alloy. Most of the initial grains are relatively large with an average size of ~300 μm and a fraction of high-angle grain boundaries of 79.2%. Cylindrical specimens with the shape of ∅10 mm × 15 mm were machined for hot compression tests.

**Figure 2:**
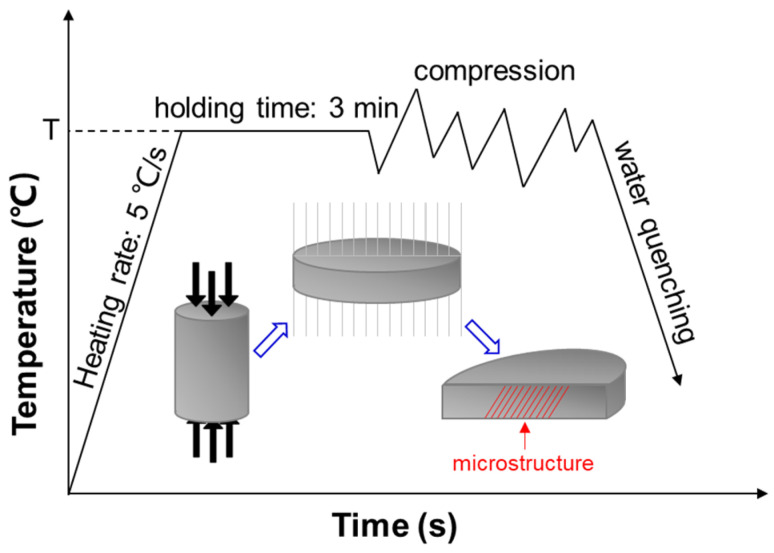
Sampling method for hot compression.

Hot compression tests were executed on a Gleeble-1500 thermomechanical simulator (DUFFERS, Poestenkill, NY, USA) at temperatures of 330, 360, 390, 420, 450, 480, and 510 °C and strain rates of 0.01, 0.1, 1, and 10 s^−1^. As the schematic diagram of the hot compression shows in Figure 2, specimens were heated to the test temperature at a heating rate of 5 °C/s and held for 3 min at the deformation temperature before compression to eliminate the gradient of temperature. The deformed specimens were water-quenched immediately after each compression test to maintain the deformed microstructure, and the simulator recorded the true stress–strain curves. The original height of the specimen was reduced until the compression reached 60% of the original height, corresponding to a strain of 0.916. To observe the microstructure, the specimen was cut along the diameter from the compression direction. The microstructure of the as-homogenized and deformed specimens was observed by JEOL JSM 7001F (JEOL, Tokyo, Japan) electron backscatter diffraction (EBSD) at 15 kV and Talos F200X G2 (Talos, Thermo Fisher Scientific, Waltham, MA, United ) transmission electron microscopy (TEM) at 200 kV. The foils used for TEM tests were polished mechanically to less than 60 μm and then punched into 3 mm disks, followed by twin-jet electropolishing, which was carried out with an electrolyte solution of ~15 V DC and a temperature below −25 °C.

## 3. Results and Discussion

### 3.1. Flow-Stress Curves

**Figure 3:**
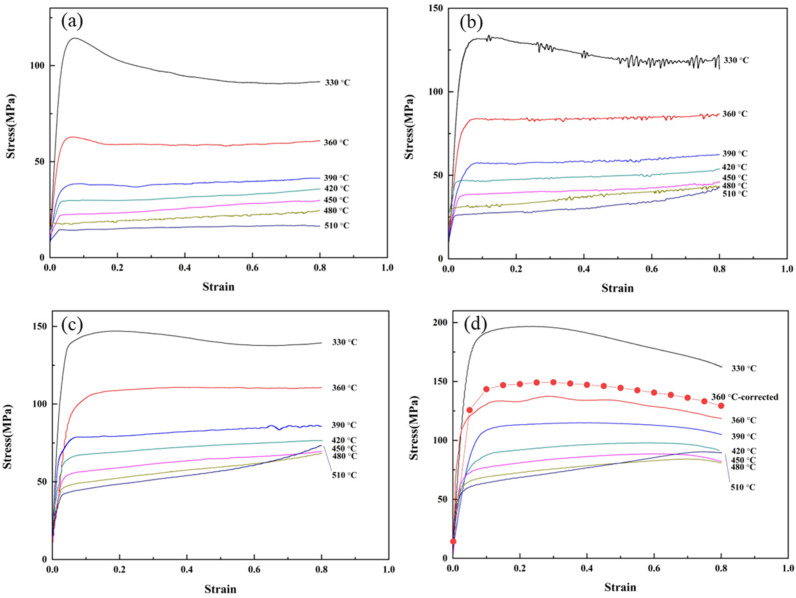
True stress–strain curves of the alloy under hot compression: (**a**) $\dot{\varepsilon }{=0.01\;s}^{-1}$, (**b**) $\dot{\varepsilon }{=0.1\;s}^{-1}$, (**c**) $\dot{\varepsilon }{=1\;s}^{-1}$, and (**d**) $\dot{\varepsilon }{=10\;s}^{-1}$ (dot line: corrected flow curve at a deformation temperature of 360 °C).

Figure 3 illustrates the true stress–strain curves of the homogenized 2198 alloy at various deformation conditions. The flow stress at the lower strain decreases obviously with the increase in deformation temperature at the same strain rate and shows an ascending tendency with the increase in strain rate, indicating that the 2198 alloy has positive strain rate sensitivity at the experimental deformation conditions.

Uniaxial isothermal hot compression is a dynamic process of work hardening and microstructure softening of the alloy. In the initial stage of hot deformation, the flow stress increases rapidly due to the increased dislocation density caused by deformation. As deformation proceeds, the thermal effect on work hardening occurs when the flow stress reaches the peak stress. When the deformation temperatures are lower than 510 °C, the dynamic softening driven by deformation energy eliminates stress concentration and decreases true stress, and then the flow stress gradually reaches the steady deformation stage. The increase in the strain rate accelerates the increment of dislocations in the alloy, which results in an increase in dislocation density and flow stress as well. The increase in deformation temperature accelerates the dislocation movement and annihilation rate, which leads to a decrease in dislocation density and flow stress. Because that dynamic softening cannot work effectively at a relatively low strain rate (0.01 s^−1^) due to the lower critical shear stress, the flow stress drops rapidly after reaching the peak stress [28]. In addition, the peak stresses are not obvious at the deformation conditions of higher temperatures because the thermal deformation behavior of the alloy is dominated by microstructure softening, including DRV and DRX. However, the flow stress values are influenced not by the dislocations, but by the precipitation, whose hindering effect on deformation is relative to its size and quantity. At higher deformation temperatures, the solid solubility of the elements involved in the precipitation increases, and the higher strain rate inhibits the coarsening of the precipitation, so the stresses grow with the increasing of strains at strain rates of 0.1, 1, and 10 s^−1^ and a deformation temperature of 510 °C, as shown in Figure 3b,c.

### 3.2. Constitutive Equation

The constitutive equation of the metallic material describes the relationship among flow stress, deformation temperature, and strain rate. The corresponding flow stress of the alloy under different deformation conditions can be predicted by the constitutive equation. The Arrhenius constitutive model was applied to establish the correlation for characterization of the flow stress behavior of the 2198 alloys, which can be expressed as Equation (1):

(1) $$\dot{\varepsilon }=A\cdot f\left(\sigma \right)\cdot \exp\left(\frac{-Q}{RT}\right)$$

where f(σ) is a function related to stress; $\dot{\varepsilon }$ denotes the strain rate (s^−1^); *T* is the deformation temperature; *Q* represents the thermal strain activation energy of hot deformation (kJ/mol), which reflects the degree of difficulty of atomic rearrangement during the deformation, and *R* is the molar gas constant (R = 8.314 J/(mol·K)).

At a low-stress level (ασ > 0.8):

(2) $$f\left(\sigma \right)=\sigma^{n_{1}}$$

At a high-stress level (ασ > 1.2):

(3) f(σ)=exp(βσ)

Within the entire stress range:

(4) $$f\left(\sigma \right)={\left[\sin h\left(\alpha \sigma \right)\right]}^{n}$$

Therefore, f(σ) can be expressed as Equation (5), which can be applied to a wide range of stress:

(5) $$\dot{\varepsilon }=A\cdot {\left[\sinh\left(\alpha \sigma \right)\right]}^{n}\cdot \exp\left(-\frac{Q}{RT}\right)$$

where *A*, *n*, α, and β are the constants related to the material (α=β/n).

The deformation activation energy of the material is different because of the different microstructural evolution during the hot deformation, as well as the material constants [29]. Assuming that the activation energy *Q* is independent of the deformation temperature T, Equations (2) and (3) are substituted into Equation (1),and logarithms are taken on both sides of the equation.

(6) $$ln\dot{\varepsilon }=lnA_{1}+n_{1}ln\sigma -Q/RT$$

(7) $$ln\dot{\varepsilon }=lnA_{2}+\beta \sigma -Q/RT$$

Figure 3 shows that when the flow stress increases rapidly, the characteristics of flow stress with the increase in strain are obviously different under different deformation conditions. Roebuck et al. [30] proposed that the barreling coefficient (*B*), which is the ratio of the volume of the specimen after deformation divided by the initial volume (the diameter is measured at the midlength of the specimen), can evaluate the effect of friction on flow stress. When *B* ≥ 1, it indicates that friction has a non-negligible effect on flow stress and must be corrected. The dimensions of specimens after deformation were measured, and the barreling coefficient B was calculated for the specimen under each deformation condition, and the values are between 1 and 1.1, indicating that the effect of friction on the flow stress of the alloy 2198 is negligible and friction correction is not necessary.

Otherwise, when the strain rate is 10 s^−1^ and the deformation temperature is 360 °C, the flow stress curve of the specimen fluctuates abnormally, and therefore the stress needs to be corrected [31]. The equation integrated by Equations (1) and (3) can be obtained:

(8) $$\dot{\varepsilon }=A\cdot \exp\left(\beta \sigma \right)\cdot \exp\left(-\frac{Q}{RT}\right)$$

Taking logarithm on both sides of Equation (8), the rearranged equation can be expressed as:

(9) $$\sigma =\frac{Q}{\beta R}\cdot \frac{1}{T}+\frac{ln\dot{\varepsilon }-lnB}{\beta }$$

The fixed strain rate σ is proportional to 1/*T*, and taking a strain of 0.3 as an example, the relationship between its measured stresses and temperature is the relationship between σ is fitted as:

(10) σ=321354.3/T−358.353

**Figure 4:**
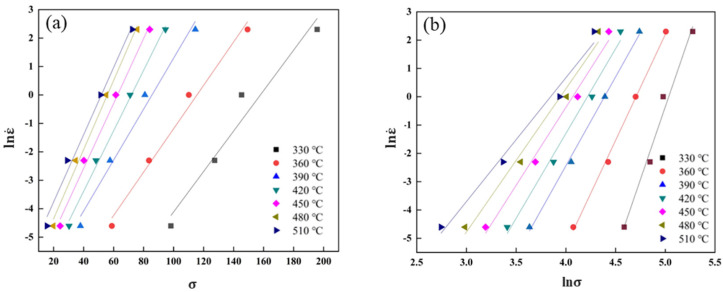
The linear relationships of ln $\dot{\varepsilon }$ versus σ and ln $\dot{\varepsilon }$ versus lnσ. (**a**) ln $\dot{\varepsilon }$ –σ, (**b**) ln $\dot{\varepsilon }$ –lnσ.

The stresses at strains from 0.00 to 0.80 with an interval of 0.05 are corrected and the dot line plotted as stress–strain curves shown in Figure 3d. As listed in Table 2, the flow stresses at the strain of 0.3 are taken as characteristic stresses to draw the ln $\dot{\varepsilon }–\sigma$ and ln $\dot{\varepsilon }$–lnσ plot and perform a linear fitting analysis. According to the slopes of the fitting lines in Figure 4a,b, it can be calculated that the material constants *n*_1_ = 6.4209 and *β* = 0.0996 and then α = *β*/*n*_1_ = 0.0155 MPa^−1^ are obtained.

The natural logarithm is taken on both sides of Equation (5) and can give the equation expressed as:

(11) $$ln\dot{\varepsilon }=lnA+nln\left[\sinh\left(\alpha \sigma \right)\right]-Q/RT$$

The expression of deformation activation energy *Q* can be obtained by calculating the partial differential of Equation (8):

(12) $$Q=R{\left[\frac{\partial \ln\dot{\varepsilon }}{\partial \ln[\sinh\left(\alpha \sigma \right)}\right]}_{T}{\left[\frac{\partial ln\left[\sinh\left(\alpha \sigma \right)\right]}{\partial \left(1/T\right)}\right]}_{\dot{\varepsilon }}$$

**Figure 5:**
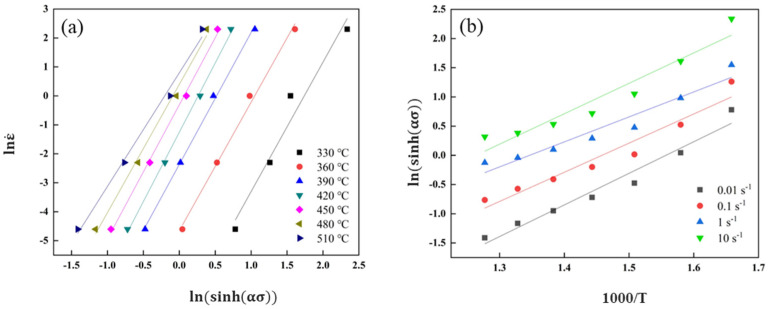
The linear relationships of ln $\dot{\varepsilon }$ versus ln[sinh(ασ)] and ln[sinh(ασ)] versus 1000/T. (**a**) ln $\dot{\varepsilon }$ –ln[sinh(ασ)], (**b**) ln[sinh(ασ)] –1000/T.

To calculate the *n* and *Q* for the present alloy, the functional relations between ln[sinh(ασ)] and $ln\dot{\varepsilon }$ are plotted and linearly fitted as shown in Figure 5a. Therefore, the *n* value is 4.4600. According to the mean slope of ln[sinh(ασ)]–1000/T fitting lines in Figure 5b, the average value of *Q* is estimated as 184.46 kJ·mol.

The relationship between flow stress and hot deformation conditions can be expressed by the Zener–Holloman (*Z*) parameter. The deformation temperature *T* and strain rate $\dot{\varepsilon }$ in Equation (5) can be transformed into a *Z* parameter related to flow stress σ, as shown in Equation (13).

(13) $$Z=A\cdot {\left[\sin h\left(\alpha \sigma \right)\right]}^{n}=\dot{\varepsilon }\cdot \exp\left(Q/RT\right)$$

The flow stress of the 2198 aluminum alloy can also be expressed by the *Z* parameter.

(14) σ=1αln{(ZA)1n+[(ZA)2n+1]12}

In order to obtain the exact value of constant *A*, Equation (13) is transformed into Equation (15) after logarithms are taken from both sides.

(15) lnZ=lnA+nln[sinh(ασ)]

**Figure 6:**
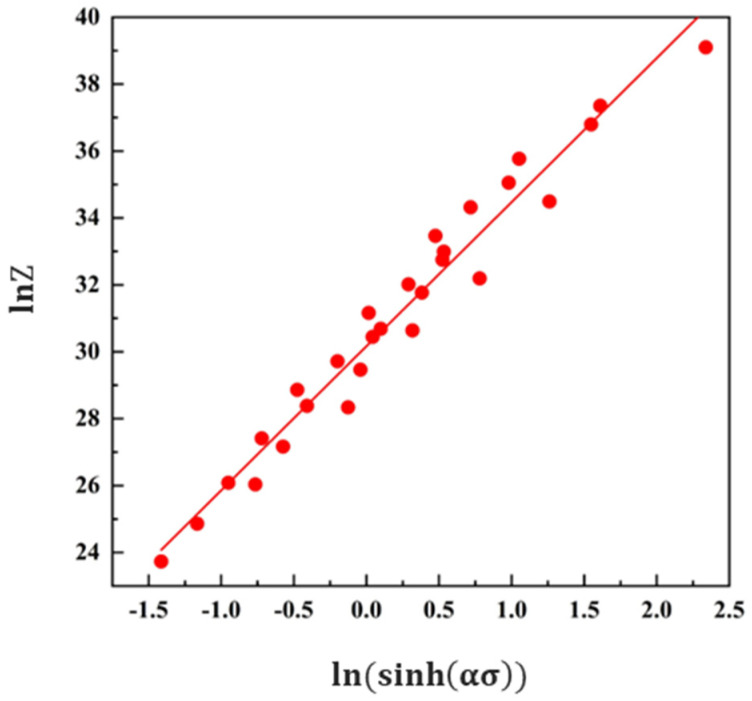
The fitting line of the Arrhenius constitutive equation.

The deformation activation energy *Q* is substituted into Equation (13), and the *Z* parameter under various deformation conditions can be calculated by the Zener–Hollomon method, as listed in Table 3. From Figure 6, the value of constant *A* can be determined according to the slope and the intercept of the fitting line. It also can be seen from Figure 6 that there is a linear relationship between ln*Z* and ln[sinh(ασ)], indicating that the relationship among flow stress, deformation temperature, and strain rate can be established by the Arrhenius relation modified by the form of hyperbolic sinusoidal. The high-temperature plastic deformation of the alloy is controlled by thermal activation. Based on the above calculation results, the constitutive equation of the 2198 aluminum alloy under hot compression can be obtained, shown as Equation (16), with the constant A of 1.259 × 10^13^ s^−1^.

(16) $$\dot{\varepsilon }=1.259\times 10^{13}{\left[\sin h\left(0.0155\sigma \right)\right]}^{4.4600}\exp\left(-1.84460\times 10^{5}/RT\right)$$

### 3.3. Establishment of Processing Map

The hot processing maps are composed of a power dissipation diagram and an instability diagram [32,33], which can reflect the microstructure evolution mechanism of the alloy under various deformation conditions and understand the thermal processability of materials. According to the dynamic material model (DMM) theory, the hot compression specimen can be regarded as an energy dissipator during the hot deformation test, and the entire amount of power given by the compressive force is mainly divided into two parts. One part of the power causes macroscopic plastic deformation in the specimen, and most of the power is switched to heat. The other part of the power is used for the microstructure evolution of materials, including DRV, DRX, and precipitation and growth of the secondary phase, which are conducive to microstructure softening. It also includes local rheology, adiabatic shear bands, and microcracks, which are not conducive to properties.

In the dynamic material model, it is assumed that during the hot compression process, the entire power of the alloy is *P*, in which the energy consumed by plastic deformation is *G*, and the dissipated energy of microstructure evolution is *J*. The relationship among *P*, *G*, and *J* is presented as follows [34]:

(17) $$P=\sigma \dot{\varepsilon }=\int_{0}^{\dot{\varepsilon }}\sigma d\dot{\varepsilon }+\int_{0}^{\sigma }\dot{\varepsilon }d\sigma =G+J$$

In the dynamic material model, it is assumed that during the hot-working process, the overall power per unit volume of the alloy is *P*, in which *G* is the energy depleted by plastic deformation, while *J* is the energy depleted by microstructure evolution.

The strain rate sensitivity index m is the ratio of the dissipation coefficient d*J* to the power dissipation rate d*G*, which is expressed as Equation(18).

(18) $$m=\frac{\partial \left(\ln\sigma \right)}{\partial \left(\ln\dot{\varepsilon }\right)}$$

The dissipator content *G* and the dissipator co-content *J* are regarded as the energy generated and consumed by plastic deformation, respectively. From the stress–strain curve, the consumption rate of dislocations is always less than or equal to the generation rate during thermal deformation. Thus, the energy loss by the microstructure evolution can be expressed as Equation(19).

(19) $$J=P-G=\frac{m}{m+1}\sigma \dot{\varepsilon }$$

For the ideal linear dissipater, *J* is equal to ideal *G*, and at this time, *m* is 1, and *J* obtains the maximum value *J_max_*:

(20) $$J_{max}=\frac{\sigma \dot{\varepsilon }}{2}=\frac{P}{2}$$

For the nonlinear dissipater, *J* < *G*, and the efficiency of the power dissipation *η* can be expressed by the ratio of *J* to *J_max_*. The dimensionless index *η* is the power dissipation efficiency and can be expressed by Equation (21).

(21) $$\eta =\frac{J}{J_{max}}=\frac{2m}{m+1}$$

According to the DMM theory, the power dissipation efficiency *η* corresponding to different strain rates and deformation temperatures can be obtained, and the isoline of power dissipation efficiency *η* of $ln\dot{\varepsilon }\;$and *T* is drawn on the power dissipation efficiency diagram. Narayana et al. [34] proposed that the criterion can be expressed by the dimensionless parameter $\xi \left(\dot{\varepsilon }\right)$, which is expressed as the following conditional formula:

(22) $$\xi \left(\dot{\varepsilon }\right)=\frac{\partial \ln\left(\frac{m}{m+1}\right)}{\partial \ln\dot{\varepsilon }}+m<0$$

When the $\xi \left(\dot{\varepsilon }\right)$ is negative, the deformation condition of the alloy is the unsafe region, corresponding to the unstable flow and the occurrence of unstable microstructure evolution of the alloy during hot compressions, such as adiabatic shear bands, flow localization, kink bands, flow rotation, and microcrack. In order to prevent the unstable deformation of the materials during hot deformation, the unstable flow zone should be avoided when determining processing parameters. Contrary to the unsafe region, there is a stable flow zone in which the parameter $\xi \left(\dot{\varepsilon }\right)$ is positive, called the safety region, where the material shows a better dynamic softening effect, such as DRV, DRX, super-plastic flow, and phase evolution. Using the rheological instability criterion corresponding to different strain rates and deformation temperature combinations under each strain, the instability and safety regions of the 2198 alloy during hot deformation are obtained.

**Figure 7:**
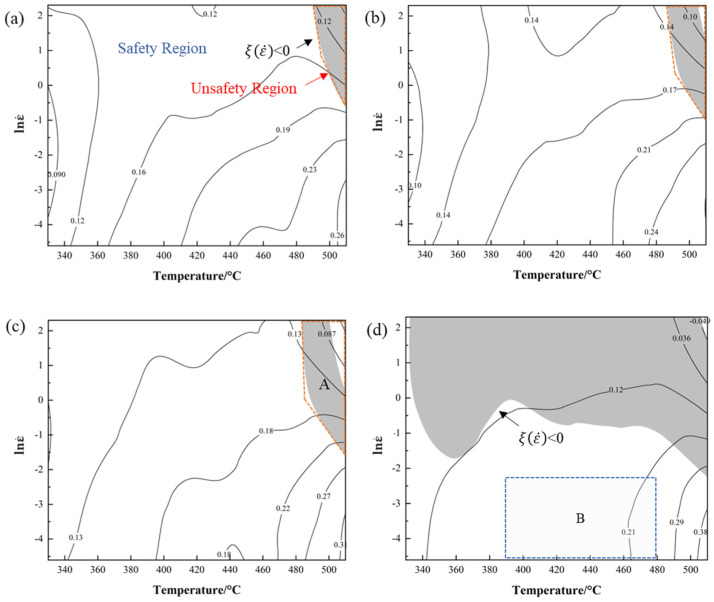
Processing maps under different strain rates: (**a**) ε = 0.2, (**b**) ε = 0.4, (**c**) ε = 0.6, and (**d**) ε = 0.8.

Figure 7 shows the hot processing maps of the 2198 alloy under different strains in the temperature range of 330~510 °C and the strain rate in the range of 0.01~10 s^−1^. The shaded area and the unshaded area in the processing maps represent the instability and the safety regions, respectively, and the number on the contours indicates the power dissipation coefficient *η*.

Figure 7 shows that the value of *η* decreases from high temperature to low temperature and from low strain rate to high strain rate, representing the decrease in the dynamic consumption of energy in the alloy. When the strain is lower than 0.4, the maximum of the power dissipation factor *η* is 0.26 due to the inadequate plastic deformation, and the unsafe region with a small area only appears in the corner of the processing maps and is concentrated in which the power dissipation factor *η* is less than 0.13, as shown in Figure 7a. When the strain rate increases to 0.6, the range of the unsafe region in the hot processing maps expands, which is reflected in region A, as shown in Figure 7c. With the increase in the strain, the area of the unsafe region begins to expand and is mainly distributed in the area with the high strain rate, where the value of maximum *η* gradually increases to 0.38. As the deformation proceeds, the density of dislocations and the deformation energy in the alloy increase, resulting in the occurrence of recrystallization and other microstructure behaviors [35]. At a strain of 0.8, the hot workability drops significantly, and the plastic unsafe region only occurs in an area with a higher strain rate and is barely affected by the deformation temperature, as shown in Figure 7d. The reason is that the low strain rate provides sufficient time for the migration of dislocations and microstructure evolutions, such as the nucleation and growth of subgrains and recrystallized grains and the dissolution, precipitation, and growth of the second phase. As the strain rate increases, the rate of dislocation proliferation is accelerated and the dislocation movement and annihilation time is shortened, resulting in a low power dissipation factor *η*.

Based on the preliminary analysis of the information in the processing maps, the relatively good processing performance corresponds to the optimal parameters of the deformation process of the 2198 Al–Li alloy, which are 390–480 °C and 0.01–0.1 s^−1^ approximately, as region B shown in Figure 7d.

### 3.4. Microstructure and Dynamic Softening Mechanism

#### 3.4.1. Flow Behavior and Microstructure Evolution

**Figure 8:**
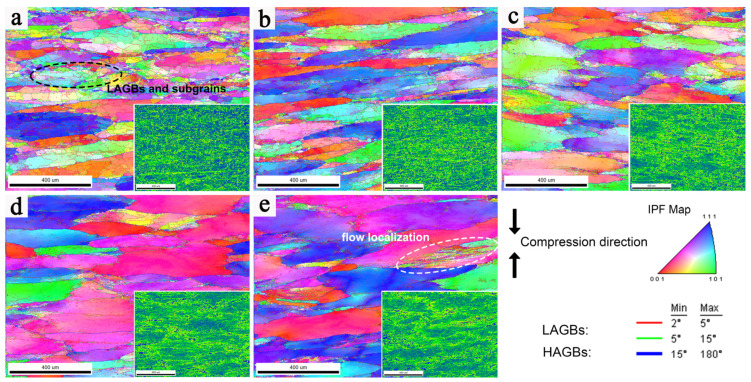
IPF image and KAM maps of the 2198 alloy hot-deformed at different conditions: (**a**) 510 °C-0.01 s^−1^, (**b**) 510 °C-0. 1 s^−1^, (**c**) 450 °C-0.1 s^−1^, (**d**) 390 °C-0.1 s^−1^, and (**e**) 390 °C-1 s^−1^.

To study the microstructure evolution of the deformed 2198 alloy during the hot compression process, the microstructure of specimens with different *Z* parameters was characterized [36]. Inverse pole figure EBSD images (IPF maps and KAM maps) overlaid with grain boundaries of the studied alloy under different hot deformation conditions are presented in Figure 8, where the red and green lines in the IPF maps indicate the low-angle grain boundaries (LAGBs, 2° < θ < 15°) and the blue lines indicate the high-angle grain boundaries (HAGBs, θ > 15°). In the KAM maps, the green area corresponds to the higher KAM value, representing the area with a higher degree of deformation, and the blue area represents the area of the newly formed grains. Figure 8a presents the region of dense LAGBs and subgrains, corresponding to the low KAM value region, where dynamic recrystallization of the grains occurs.

Furthermore, the deformation conditions in Figure 8a–d are located in the safety region of the processing maps, and those in Figure 8e are located in the unsafe region of the processing maps. The EBSD images show that the initial grains are broken and elongated along the direction perpendicular to the compression direction during the hot deformation.

At low *Z* values, the grain boundaries of deformed grains are relatively clear and smooth with uniform distribution, while at high *Z* values, the distribution of deformed grains is more concentrated and uneven, where the deformed microstructure shows severe flow localization phenomenon caused by shear band concentration. The adiabatic shear band will easily form during the deformation with the increase in local temperature and then promote the flow localization. Under the deformation conditions with a high *Z* value, the deformation time is too short to make the heat dissipate in time, which is more likely to cause a local temperature concentration, resulting in flow localization. Moreover, with the further increase in the *Z* value, the phenomenon of flow localization becomes more obvious. In addition, the uneven and insufficient dynamic softening is also the reason for the formation of flow localization, where the thickness of banded deformed grains is smaller and broken obviously. It indicates that the deformation is severer in the area of flow localization and leads to an increase in the deformation resistance of the alloy. Therefore, the more serious flow localization at a high *Z* value is also the reason for higher flow stress.

**Figure 9:**
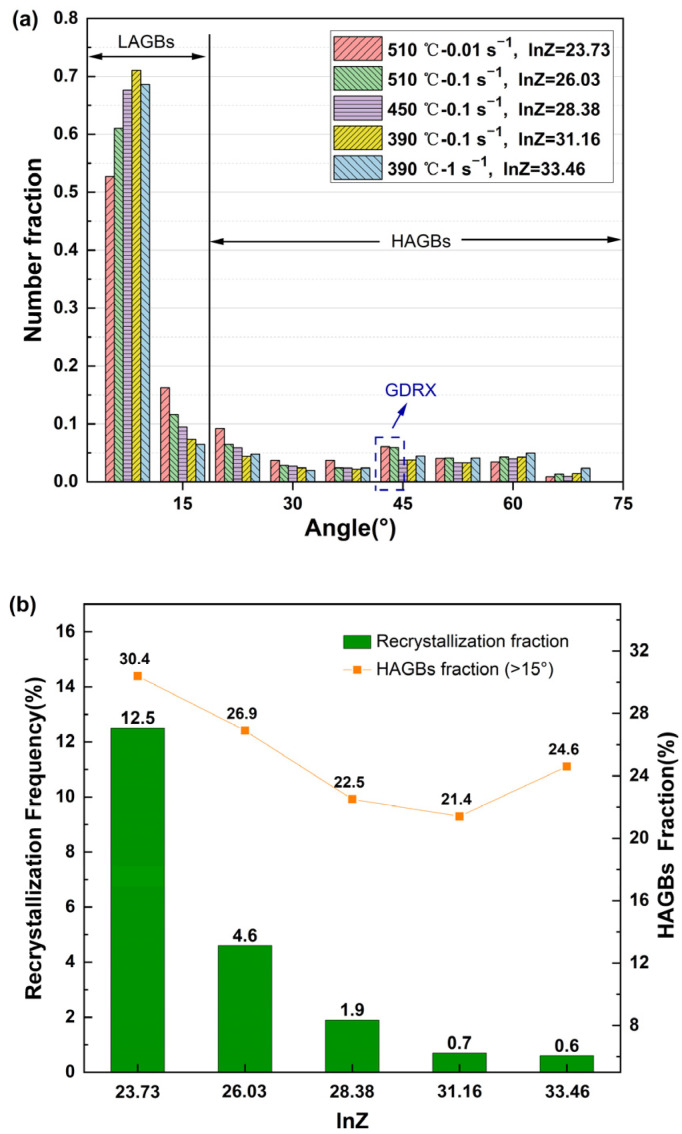
(**a**) Distribution of boundary misorientation angle of specimens and (**b**) the recrystallization frequency of alloy at different deformation conditions.

The distribution of the boundary misorientation angle of the 2198 alloy with different ln*Z* is presented in Figure 9a. The fraction of HAGBs gradually increases with a decrease in ln*Z*. With the decrease in ln*Z*, which represents the decrease in deformation rate or the increase in deformation temperature, the fraction of LAGBs tends to decrease gradually. The LAGB fraction reflects the dynamic softening ability of the alloy, and the LAGBs indicate that most of the dislocations disappear through climbing or rearrangement, resulting in the decrease in the deformation energy and resistance. However, because the location of the deformation condition of 390 °C/1 s^−1^ is at the unsafe region in the processing map, the fraction of LAGBs does not conform to the law of change [37]. Because the deformation of microstructure in the unsafe region is severe, fragmented grains form at the triangular region between the deformed grains, increasing the local deformation distortion energy and the fraction of LAGBs caused by static recrystallization, and even possibly forming shear bands [38]. Due to the higher stacking fault energy of the aluminum alloy, it is difficult for the occurrence of DRX at low deformation temperatures, and the equilibrium between DRV softening and work hardening is mainly involved in the deformation process. As shown in Figure 9b, with the decrease in ln*Z* values, based on increasing deformation temperature or decreasing strain rate, the frequency of recrystallization increases, which corresponds to the increase in HAGB fractions. Contrary to the low ln*Z*, a lot of LAGBs are formed inside the deformed grains, and the subgrains have complete profiles with a smaller size compared with the DRX grains [39].

**Figure 10:**
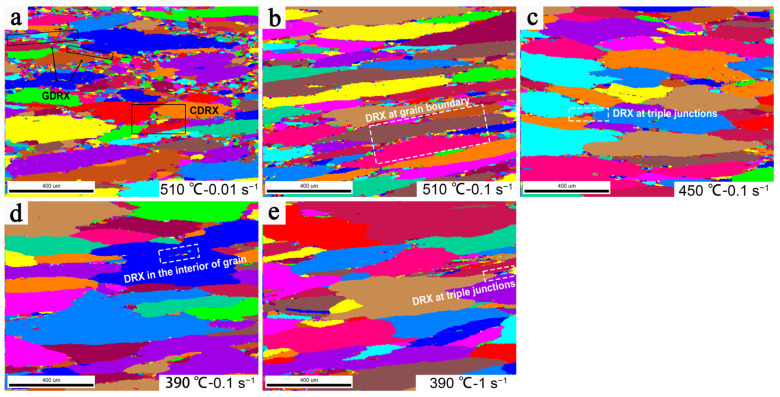
Unique grain color maps of the 2198 alloy hot-deformed at various conditions: (**a**) ln*Z* = 23.73, (**b**) ln*Z* = 26.03, (**c**) ln*Z* = 28.38, (**d**) ln*Z* = 31.16, and (**e**) ln*Z* = 33.46.

The microstructures of the deformed 2198 alloy were composed of deformed grains, newly formed equiaxed grains, and subgrains. According to the fine-equiaxed grains along the serrated grain boundaries shown in Figure 10, it can be deduced that DRX occurs in the deformed alloy. DRX occurs in the microstructure under different studied deformation conditions, but the content and distribution location are different. DRX is mostly concentrated at the triangular region surrounded by deformed grains as the marked rectangular area, and more dynamic recrystallized grains are gradually formed along the grain boundaries and in the interior of some grains with the decrease of ln*Z*. The DRX effect is limited under the deformation conditions with high ln*Z*, but when the ln*Z* value is high, the region where the deformed microstructure has obvious flow localization is also recrystallized along the grain boundary, as revealed in Figure 10e.

The DRX mechanisms differ in the interior of grains or at the grain junctions, and they may also be inconsistent near grain boundaries. It is necessary to distinguish the type of recrystallization mechanisms. There are mainly three dynamic recrystallization mechanisms: DDRX, CDRX, and GDRX. DDRX has a typical process of nucleation and growth of recrystallized grains and is also related to the gradual accumulation of dislocations [40,41]. CDRX is accompanied by the changes from low-angle grain boundaries to high-angle grain boundaries, as the grains and subgrains shown in Figure 9a and Figure 10a, where the transformation of grain boundary orientation can be observed clearly. GDRX is due to the fragmentation of deformed grains. The original grain boundaries, especially the triangular grain boundaries, are the ideal position for the nucleation of DDRX, while GDRX is related to broken grains caused by the contact of jagged bow-out grain boundaries, which is also mainly distributed near the grain boundaries [42]. However, there are still differences between them, because the GDRX mechanism is accompanied by the deformed grains being broken perpendicular to the compression direction, and the grain size is small, which is generally 2 to 3 times the size of subgrains [43,44]. For example, Figure 10a shows that there are obvious new grains with the same orientation produced by the fragmentation of deformed grains along the elongation direction. With the elongation and narrowing of the original grain during deformation, the HAGBs will form through the mechanism of GDRX, and the boundary misorientation angle will show a bimodal distribution [45]. The fraction of HAGBs increases obviously when the values of ln*Z* are 23.73 and 26.03, as shown in Figure 9a.

In summary, the average grain size of dynamic recrystallization decreases with the increase in ln*Z*, and the recrystallization fraction decreases, which indicates that the nucleation and growth of dynamic recrystallization decrease correspondingly. Under the deformation conditions with a low ln*Z* value, the CDRX and GDRX mechanisms work in the deformed microstructure. The migration ability of the dislocations and grain boundaries is sensitive to deformation temperature. At high deformation temperatures, the migration speed of dislocations is faster, and HAGBs are more likely to bow out and migrate to form discontinuous dynamic recrystallization nuclei, which also grow faster. Under the deformation conditions with a high ln*Z* value, there is a certain number of DDRX grains in the alloy. By controlling DRV and DRX during hot working, the substructure of the alloy is strengthened, and the mechanical properties of the material are improved. Moreover, hot working includes forging, extrusion, and rolling, and the strain rates of them are generally in the range of 0.05–5 s^−1^ in industrial production. Based on the further analysis supported by microstructure, the optimal ln*Z* value of the 2198 Al–Li alloy is 23.73–31.16, which corresponds to the parameters of the deformation process around 450–480 °C approximately.

#### 3.4.2. The Influence of Deformation Temperature and Strain Rate on Microstructure

**Figure 11:**
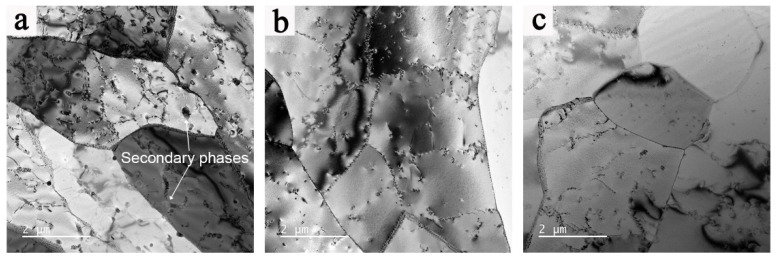
TEM images of the 2198 alloy hot-deformed at different temperatures: (**a**) 390 °C/0.1 s^−1^, (**b**) 450 °C/0.1 s^−1^, and (**c**) 510 °C/0.1 s^−1^.

TEM was used to study the influence of different deformation temperatures and strain rates on dislocation behavior and submicrostructure characteristics. Figure 11 shows the TEM images of specimens deformed at different deformation temperatures, and the microstructure of the specimen shows fine polygonal grains with high-density dislocations in the interior of grains, which conflicts with the DRX mechanism. With the increase in deformation temperature, the grain size increases accompanied by the decrease in dislocation density, which is attributed to the enhancement of thermal deformation energy provided by the high temperature and sufficient time for the migration of dislocations. Moreover, a certain number of secondary phases was observed in the matrix shown in Figure 11a, which enhances the pinning effect on dislocations and leads to the increase in dislocation density [46].

**Figure 12:**
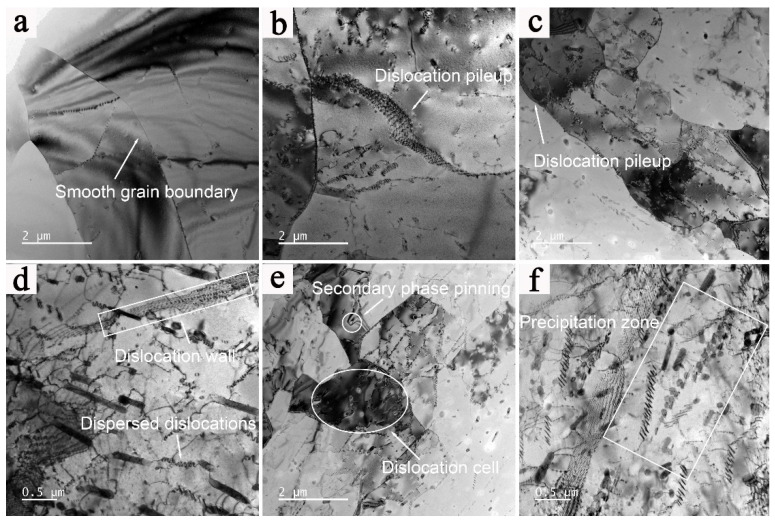
TEM images of the 2198 alloy hot-deformed at different strain rates: (**a**) 510 °C/0.01 s^−1^, (**b**) 510 °C/0.1 s^−1^, (**c**,**d**) 390 °C/0.1 s^−1^, and (**e**,**f**) 390 °C/1 s^−1^.

Figure 12 shows the TEM images of specimens deformed at different strain rates. At a given deformation temperature, it can be easily found that the strain rate has an obvious influence on the microstructure evolution. Under the deformation condition of 510 °C/0.01 s^−1^, the subgrain form with a low density of intragranular dislocations owing to the climbing and cross-slipping of dislocations, as shown in Figure 10a. At such a low strain rate, the large-sized DRX grains gradually form based on the consolidated subgrains, and the grain boundaries are straight and smooth without obvious dislocation entanglement. When the strain rate increases to 0.1 s^−1^, the dispersed distribution of dislocations can be observed inside the deformed grains because the time for the migration of strain-induced dislocations is shortened [47], and the annihilation and transformation of dislocations are restrained, resulting in the formation of subgrain structure characteristics with dislocation pile-up, as shown in Figure 12b,c. Figure 12c–f present the TEM images of deformed specimens with different strain rates at lower deformation temperatures for further analysis. The density of dislocations increases significantly, and there is a certain amount of dislocation intersection and entanglement in the grains. The dislocation movement is impeded by the secondary phase, forming tangled dislocations and dislocation cells, as shown in Figure 12e. At a strain rate of 1 s^−1^, there is a slight decrease in the overall density of dispersed dislocation inside the grains and an increase in the dislocation wall due to the slow rearrangement of dislocations, as shown in Figure 12e,f. At deformation conditions of 390 °C/0.1 s^−1^ and 390 °C/1 s^−1^, precipitation zones can be observed in the interior of grains and at grain boundaries clearly, hindering grain boundary migration and inhibiting the formation of DRX. The secondary phases may also be the reason that DRX does not occur easily under the deformation conditions of 2198 Al−Li alloys with a lower deformation temperature and higher strain rate. It is hard to observe the existence of secondary phases at a temperature of 510 °C.

## 4. Conclusions

In this work, the hot deformation behavior of the 2198 alloy and microstructure evolution were investigated based on the flow stress curves obtained through hot compression tests at various deformation temperatures and strain rates. The main results can be summarized as follows:

(1) The flow stress of the 2198 Al–Li alloy during the hot compression test increases with the increase in strain in the initial stage of deformation, and then gradually tends to be stable due to the increasing effect of microstructure softening. In general, the characteristic stress is inversely proportional to the deformation temperature and proportional to the strain rate.
(2) The Arrhenius constitutive equation of flow behavior for the 2198 Al–Li alloy was determined as $\dot{\varepsilon }=1.259\times 10^{13}{\left[\sin h\left(0.0155\sigma \right)\right]}^{4.4600}\exp\left(-1.84460\times 10^{5}/RT\right)$. The scope of the unsafe region in the processing maps expands with the increase in strain, and the optimal parameters of the deformation process of the 2198 Al–Li alloy is around 450–480 °C approximately, providing efficient guidance for industrial processing, such as forging, extrusion, and rolling.
(3) The CDRX and GDRX mechanisms mainly occur at low *Z* values, corresponding to the conversion of the misorientation angle of grain boundaries from LAGBs into HAGBs, while the DDRX mechanism mainly occurs at moderate and high *Z* values.
(4) At low deformation temperatures and high strain rates, flow localization occurs at the triple junctions of deformed grains. The density of secondary phases increases with the increase in the *Z* value, constraining the forming of DRX grains by hindering the migration of grain boundaries.

## Figures and Tables

**Table 1:** Chemical composition of 2198 Al–Li alloy (wt.%).

Element	Cu	Li	Mg	Ag	Zr	Mn	Ti	Al
wt.%	3.07	0.92	0.42	0.26	0.12	0.03	0.03	Bal.

**Table 2:** Characteristic stresses under different deformation conditions (MPa).

	Strain Rate/s^−1^	0.01	0.1	1	10
Temperature/°C					
330		98.20	127.37	145.35	195.72
360		58.85	83.57	110.16	149.32
390		37.87	57.60	80.86	114.49
420		30.27	48.24	71.00	94.57
450		24.36	40.19	61.48	84.10
480		19.81	34.65	55.02	75.83
510		15.55	29.04	51.30	72.43

**Table 3:** ln*Z* values of the 2198 alloy with different strain rates and temperatures.

Temperature (°C)	Strain Rate (s^−1^)			
	0.01	0.1	1	10
330	32.19	34.49	36.79	39.10
360	30.44	32.75	35.05	37.35
390	28.86	31.16	33.46	35.77
420	27.41	29.71	32.02	34.32
450	26.08	28.38	30.69	32.99
480	24.86	27.16	29.46	31.77
510	23.73	26.03	28.34	30.64

## Data Availability

The data presented in this study are available on request from the corresponding author.
